# The formation and design of the TRIAGE study - baseline data on 6005 consecutive patients admitted to hospital from the emergency department

**DOI:** 10.1186/s13049-015-0184-1

**Published:** 2015-12-01

**Authors:** Louis Lind Plesner, Anne Kristine Servais Iversen, Sandra Langkjær, Ture Lange Nielsen, Rebecca Østervig, Peder Emil Warming, Idrees Ahmad Salam, Michael Kristensen, Morten Schou, Jesper Eugen-Olsen, Jakob Lundager Forberg, Lars Køber, Lars S. Rasmussen, György Sölétormos, Bente Klarlund Pedersen, Kasper Iversen

**Affiliations:** Department of Cardiology, Endocrinology and Nephrology, North Zealand Hospital, Copenhagen University Hospital, Copenhagen, Denmark; Department of Cardiology, Herlev Hospital, Copenhagen University Hospital, Copenhagen, Denmark; Clinical Research Centre, Copenhagen University Hospital Hvidovre, Copenhagen, Denmark; Emergency Department, North Zealand Hospital, Copenhagen University Hospital, Copenhagen, Denmark; Department of Cardiology, Rigshospitalet, Copenhagen University Hospital, Copenhagen, Denmark; Department of Anaesthesia, Centre of Head and Orthopaedics, Rigshospitalet, University of Copenhagen, Copenhagen, Denmark; Department of Clinical Biochemistry, North Zealand Hospital, Copenhagen University Hospital, Copenhagen, Denmark; Centre of Inflammation and Metabolism (CIM) and Centre for Physical Activity Research (CFAS), Rigshospitalet, Copenhagen University Hospital, Copenhagen, Denmark

**Keywords:** Triage, Biomarkers, Emergency department risk stratification, Avoidable hospitalization, Biobank

## Abstract

**Background:**

Patient crowding in emergency departments (ED) is a common challenge and associated with worsened outcome for the patients. Previous studies on biomarkers in the ED setting has focused on identification of high risk patients, and and the ability to use biomarkers to identify low-risk patients has only been sparsely examined. The broader aims of the TRIAGE study are to develop methods to identify low-risk patients appropriate for early ED discharge by combining information from a wide range of new inflammatory biomarkers and vital signs, the present baseline article aims to describe the formation of the TRIAGE database and characteristize the included patients.

**Methods:**

We included consecutive patients ≥ 17 years admitted to hospital after triage staging in the ED. Blood samples for a biobank were collected and plasma stored in a freezer (−80 °C). Triage was done by a trained nurse using the Danish Emergency Proces Triage (DEPT) which categorizes patients as green (not urgent), yellow (urgent), orange (emergent) or red (rescusitation). Presenting complaints, admission diagnoses, comorbidities, length of stay, and ‘events’ during admission (any of 20 predefined definitive treatments that necessitates in-hospital care), vital signs and routine laboratory tests taken in the ED were aslo included in the database.

**Results:**

Between September 5^th^ 2013 and December 6^th^ 2013, 6005 patients were included in the database and the biobank (94.1 % of all admissions). Of these, 1978 (32.9 %) were categorized as green, 2386 (39.7 %) yellow, 1616 (26.9 %) orange and 25 (0.4 %) red. Median age was 62 years (IQR 46–76), 49.8 % were male and median length of stay was 1 day (IQR 0–4). No events were found in 2658 (44.2 %) and 158 (2.6 %) were admitted to intensive or intermediate-intensive care unit and 219 (3.6 %) died within 30 days. A higher triage acuity level was associated with numerous events, including acute surgery, endovascular intervention, i.v. treatment, cardiac arrest, stroke, admission to intensive care, hospital transfer, and mortality within 30 days (*p* < 0.001).

**Conclusion:**

The TRIAGE database has been completed and includes data and blood samples from 6005 unselected consecutive hospitalized patients. More than 40 % experienced no events and were therefore potentially unnecessary hospital admissions.

**Electronic supplementary material:**

The online version of this article (doi:10.1186/s13049-015-0184-1) contains supplementary material, which is available to authorized users.

## Background

There are annually around 800.000 acute admissions of adults in Denmark, and numbers have increased in recent years [1]. Crowding in emergency departments (ED) and hospital wards are common problems and can be detrimental to patient outcome [2–4]. It has recently been estimated that over 20 % of all acute hospital admissions are potentially avoidable [5]. Discovering ways of reducing unnecessary hospitalization is essential, as hospital admission is associated with adverse events such as infections, accidental injuries and cardiovascular complications [6], and early discharge has been shown to improve outcome [7, 8].

Triage algorithms for stratifying patients in the ED according to acuity level have been developed and employed for the purpose of prioritizing ressources and ensuring adequate attention to the sickest patients [9]. The present triage algorithms have not been designed to identify patients in the ED with such a low need of acute treatment, that they can be immediately discharged to an outpatient clinic or follow-up by their own general practitioner.

New efficient tools enabling identification of these patients could result in substantial economical savings [10], provide better time to treat the high-risk patients and probably reduce adverse events and inconvenience associated with unnecessary hospitalizations of low-risk patients.

Several old and new biomarkers are useful to estimate short term prognosis [11–16] and a recent theoretical study has suggested that the use of biomarkers would substantially strengthen the existing triage algorithms [17].

The TRIAGE study is designed primarily to examine if a system combining biochemical markers with currently available data, including observations by a triage nurse, can identify patients who can be safely discharged to outpatient follow-up. Secondary objectives of the TRIAGE study are to investigate the prognostic value of several new and old biomarkers in a large population of unselected and consecutively admitted patients. These primary and secondary aims will be assesed in future publications.

The aims of the present article are to describe the formation of the TRIAGE database, the design of the study, and the characteristics of the included patients.

## Methods/Design

The formation of the database for the TRIAGE study took place at North Zealand University Hospital, which is a large hospital in the Capital Region of Denmark. The hospital is a 24-h acute care hospital offering medical, surgical, level-2 trauma, emergency and intensive care services for 310 000 citizens in North Zealand [18].

### Study design

The TRIAGE study was prospective and observational. There was non-stop inclusion of consecutive patients presenting to an emergency department (24 h 7 days/week) until target number was met (=6000). Admission, and thereby inclusion in the study, was defined as refferal to a bed and blood samples drawn in the ED. We included all patients admitted through the emergency department, but patients ≤ 17 years and obstetric patients were not included since they were admitted directly to the paediatric ED or obstetrics department, respectively. Patients detected in the field with major trauma, ST-elevation acute myocardial infarction or stroke within 2–3 h were admitted to the tertiary centre in the region.

### Data collection

All data were merged using the unique personal CPR number (‘Central Personal Registry’) into a secure web-based database, except the biomarker results. The biomarkers will be registered in an additional database (blinded and keyed by a subject identifyer). After the databases have been completed, all data will be merged and transferred to a central public server in Statistics Denmark and blinded prior to further analyses. Ongoing information about death, hospitalization and medication can be retreived from these central servers. The data derive from the three different sources listed below.

#### Triage data

Data were obtained by trained and experienced triage-nurses at the time of admission and entered into the database by a medical student and included age, gender, vital signs and triage category. Table 1 shows the five-level Danish Emergency Proces Triage (DEPT) used in the ED, patients are categorised into five triage levels based on vital signs and a presenting complaint algorithm [19]. DEPT is a Danish adaption and modification of the “Adaptive Process Triage” (ADAPT) developed in Sweden in 2006 [20]. Patients triaged blue were not included in the study as they were never admitted at North Zealand Hospital according to our definition. Heart rate (HR), blood pressure (BP), respiratory rate (RR) and peripheral arterial oxygen saturation (SpO_2_) were measured on a patient monitor (Phillips Intellivue MP30) and temperature (Tp) was measured with an ear thermometer (Covidien Genius™2).

**Table 1:** The five categories of DEPT triage

Rescuscitation	Emergent	Urgent	Non-urgent	Non-urgent
Life threatened	Critical illness	Potentially unstable	Stable	Unaffected
Constant re-evaluation	Re-evaluation every 15 min.	Re-evaluation every 60 min.	Re-evaluation every 180 min.	Re-evaluation every 240 min.
Acute Team	Physician and Nurse	Nurse → physician*	Nurse → Physician*	Nurse or physician

DEPT (Danish Emergency Process) Triage: Each patient is assigned a triage level for each of the two main descriptors: 1) Vital signs and 2) presenting complaint. The nurse performing triage can increase a patients triage level if she believes the patient is more ill than what is determined by DEPT [19]. *Patients are seen by a physician, but the evaluation can be made by a nurse initially

#### Blood sample data

Blood samples were drawn by either a phlebotomist or a medical student as soon as possible (within 0–60 min following admission) and the procedure followed a predefined sequence to minimize the risk of preanalytical errors [21].

Results from routine laboratory examinations were later retrieved from the hospital database (LABKA ll. Version 2.5.0.H2 Computer Sciences Corporation (CSC)) including CRP, hemoglobin, sodium, potassium, creatinine, albumin (Dimension Vista® 1500, Siemens Medical Solutions Diagnostics), and white blood cell count as well as platelet count (Sysmex XE-5000, Sysmex Corporation).

Additional blood was collected for storage in the biobank and subsequent analysis of a wide range of new inflammatory biomarkers. Blood samples were drawn simultaneously with the routine laboratory tests, and they were spun for 10 min at 1800 G within 120 min and transferred to 13 different cryotubes (8 serum, 4 plasma and 1 buffycoat per patient), each tube containing 0.5-1 ml. The cryotubes were immediately frozen at −20 °C and transferred to a central −80 °C freezer on the following day for final storage. The laboratory procedures were performed by medical students, who had been carefully instructed prior to the study.

#### Data from the patient file

Data on comorbidity, smoking habits, alcohol intake, social status, events during hospitalization (any of 20 predefined definitive treatments that necessitates in-hospital care, i.e., Acute surgery, endovascular procedure, death within 30 days etc. - see Table 3. The events had been predefined in the study protocol), hospital length of stay (LOS), and hospital discharge diagnosis were also retrieved from the patient file (OPUS Arbejdsplads, version 2.5.0.0 Computer Sciences Corporation (CSC)). Information about death within 30 days after admission was retrieved from the central public server in Statistics Denmark.

### Identification of unnecessary admissions

An unnecessary hospitalization was defined as a situation where a patient could have been handled by the general practioner or by an outpatient clinic within 1–2 weeks. Patients who experienced at least one of the predefined events, were identified and categorized as definate necessary hospitalizations, but the list of events was not constructed to deem whether a hospitalization could have been unnessesary as it has previously been argued that a list of predefined criteria for measuring the appropriateness of an admission can never be sufficient [22]. Therefore we had two specialists in internal medicine or emergency medicine independently assessing the files of patients with none of the specified events to determine if it could be considered an unnecessary or necessary admission. Only when both specialists agreed, the admission was considered unnecessary. The specialists were blinded from the information of whether they were the primary or secondary reviewer of the patient case and from the judgement of the other.

### Identification of high-risk patients

Patients with admission to intensive care unit within 30 days or semi-intensive care unit within 14 days or death within 30 days will be categorized as high-risk patients.

### Ethics

The study was conducted according to Danish ethical regulations and was approved by the Danish Data Protection agency (J. 2007-58-0015).

### Consent

Regarding consent from patients, formal ethical approval was not necessary for this study. After completion of the biobank, all data will be transferred to a central server. All patients will thereafter be blinded, but with a unique patient number. It is thereafter possible to connect the triage database with all other Danish registers and get information of death, biochemistry, medication, admissions etc. This procedure is in accordance with ethical regulations.

### Sample size

A definite sample size was not caluclated prior to the study, as the percentage of patients with unnecessary admission were unknown. We anticipated that at least 20 % of the population would be unnecessarily admitted. A triage used for sending patients home directly from the ED should ensure that the risk of it being a necessary admission should be very small (less than 0.1 % corresponding to a specificity of 99.9 %). The sensitivity of the model should be at least 20 %. Thus, with 6000 admissions the triage should be able to identify 240 (20 %) of the hypothesized 1200 unnecessary admissions, that could be discharged immediately.

### Statistics

Continuous data are presented as mean with standard deviation (SD) or as median with interquartile range (IQR) where appropriate and categorical data are presented as n(%). Continuous variables are compared using the Student’s *t*-test and categorical variables are compared using the Chi-square-test. One-way ANOVA or Kruskal Wallis H test are used to compare continuous data across the 4 triage categories when data is normally or non-normally distributed, respectively. Reporting frequencies of events by different investigators were compared using chi-squared tests. were A two-sided probability of *p* < 0.05 was considered statistically significant. All analyses were performed using SPSS software version 22.0 (IBM corp., USA).

## Results

**Fig. 1:**
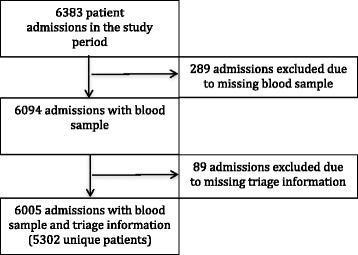
The selection of patient admissions included in the study. *Secondary admissions ranged from 2–12 readmissions

We included data on 6005 consecutive patient admissions between September 5th, 2013 and December 6th, 2013. This corresponds to 94.1 % of the total number of admitted patients (n = 6383) in the study period (Fig. 1).

**Fig. 2:**
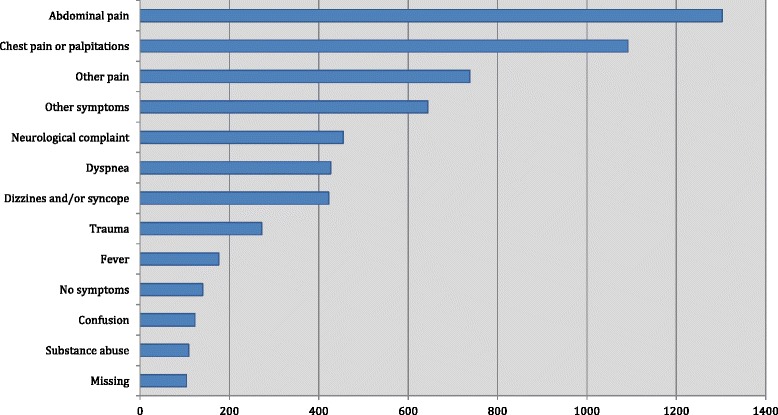
The patient admissions grouped within’Presenting complaints’. ‘Abdominal pain’ included diarrhea, nausea, melena and hematemesis. ‘Other’ included: Lower urinary tract symptoms, abscess, allergic reactions

**Fig. 3:**
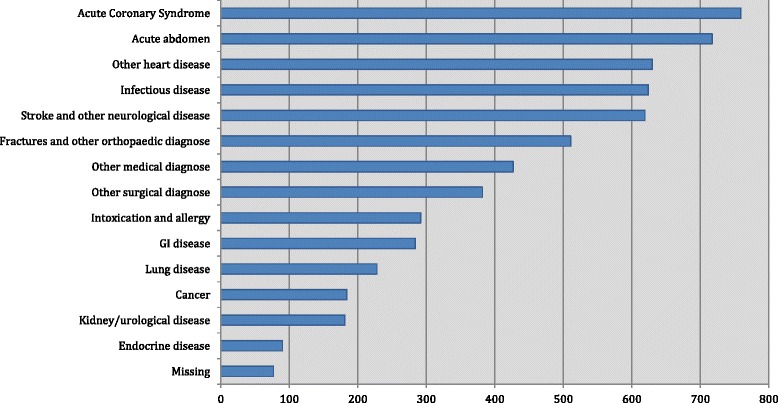
Patient admissions grouped within ‘Referral diagnoses’ ‘Other surgical diagnose’ included: Abscess, cysts, hernia, hemorrhoid, hematemesis. ‘Other medical diagnose’ included: Dehydration, Anaemia, Dizziness, Syncope and Social factors

Median age was 62 years (IQR 46–76) and 49.8 % were males. Median LOS was 1 day (IQR 0–4). Mean vital values were: systolic BP 136 mmHg (SD 23.5), HR 82 per min (SD 19), Tp 36.8 °C (SD 0.8), SpO_2_ 97.7 % (SD 2.5) and RR 17 per min (SD 3.8). There were 570 patients (9.5 %) who recieved oxygen therapy during SpO_2_ measurement. Vital values according to triage stage are shown in Additional file 1: Table S1. A total of 2658 patients (44.2 %) experienced no events during admission, while 158 (2.6 %) were admitted to intensive or intermediate-intensive care unit and 219 (3.6 %) died within 30 days. Distribution of patient presenting complaints and admission diagnoses are found in Figs. 2 and 3, respectively.

Patient characteristics according to triage category are displayed in Table 2. There was a significant relationship between level of urgency and increasing age, male gender, and comorbidity. There was a significant association between triage category and numerous events during hospitalization, including acute surgery, endovascular intervention, i.v. treatment, cardiac arrest, stroke, admission to intensive care, transfer to another hospital, and mortality within 30 days (Table 3).

**Table 2:** Demographics and comorbidities according to Triage stage (unique patients)

	Green (*n* = 1741)	Yellow (*n* = 2094)	Orange (*n* = 1445)	Red (*n* = 22)	*P* value*
Age, median years (IQR)	61 (44–76)	61 (44–76)	64 (50–77)	72 (66–82)	<0.001
Gender, male n (%)	825 (47.4)	1015 (48.5)	747 (51.7)	17 (77.3)	0.004
LOS, median days (IQR)	1.0 (0–3)	1.0 (0–4)	1.0 (1–5)	5.0 (1.75-8)	<0.001
Ischemic heart disease n (%)	109 (6.3)	150 (7.2)	223 (15.4)	4 (18.2)	<0.001
Heart failure, n (%)	54 (3.1)	102 (4.9)	112 (7.8)	5 (22.7)	<0.001
Hypertension, n (%)	352 (20.2)	420 (20.1)	400 (27.7)	7 (31.8)	<0.001
Diabetes, n (%)	171 (9.8)	189 (9.0)	187 (12.9)	5 (22.7)	<0.001
COPD, n (%)	96 (5.5)	171 (8.2)	131 (9.1)	4 (18.2)	<0.001
Kidney disease, n (%)	56 (3.2)	75 (3.6)	49 (3.4)	1 (4.5)	0.925
Liver disease, n (%)	29 (1.7)	31 (1.5)	15 (1.0)	2 (9.1)	0.010
Rheumatic disease, n (%)	34 (2.0)	47 (2.2)	26 (1.8)	0 (0.0)	0.709
Cancer, n (%)	248 (14.2)	292 (13.9)	179 (12.4)	2 (9.1)	0.389
Smoking, n (%):					<0.001
Former	314 (18.0)	456 (21.8)	381 (26.4)	5 (22.7)	
Current	405 (23.3)	488 (23.3)	368 (25.5)	8 (36.4)	
Alcohol abuse, n (%)	143 (8.2)	156 (7.4)	109 (7.5)	5 (22.7)	0.052
Social aspects, n (%):					
Living alone	352 (20.2)	396 (18.9)	341 (23.6)	3 (13.6)	0.006
Domestic help	153 (8.8)	160 (7.6)	147 (10.2)	2 (9.1)	0.075
Nursing home	77 (4.4)	107 (5.1)	67 (4.6)	1 (4.5)	0.788

*P value indicates significant difference between the triage categories. Alcohol abuse was defined as ≥ 3 units of alcohol/day

*LOS* Lenght of stay, *COPD* Chronic obstructive pulmonary disease. Green/yellow/orange/red refers to triage acuity level in DEPT: non-urgent/urgent/emergent/resuscitation respectively, (Table 1 for detalis)

**Table 3:** Distribution of ‘events’ according to Triage stage

Panel A - Events qualifying for a necessary admission	Green (*n* = 1978)	Yellow (*n* = 2386)	Orange (*n* = 1616)	Red (*n* = 25)	*p*-value*
No events, n (%)	904 (45.7)	1087 (45.6)	663 (41.0)	0 (0)	<0.001
Acute surgery, n (%)	51 (2.6)	71 (3.0)	80 (5.0)	4 (16)	<0.001
Surgery during admission, n (%)	125 (6.3)	135 (5.7)	115 (7.1)	2 (8)	0.305
Non-scheduled surgery within 14 days, n (%)	37 (1.8)	46 (1.9)	37 (2.3)	2 (8)	0.145
Trombolysis, n (%)	6 (0.3)	11 (0.5)	11 (0.7)	0 (0)	0.415
Endovascular procedure, n (%)	38 (1.9)	50 (2.1)	133 (8.3)	2 (8)	<0.001
Antibiotics > 24 h within 7 days, n (%)	367 (18.5)	575 (24.1)	284 (17.6)	9 (36)	<0.001
i.v.-treatment with diuretics > 1 times within 14 days, n (%)	36 (1.8)	84 (3.5)	73 (4.5)	12 (48)	<0.001
Other i.v.-treatment > 1 times within 14 days, n (%)	414 (20.9)	487 (20.4)	318 (19.7)	14 (56)	<0.001
Admissions lasting more than 3 days, not due to social factors or complications due or to initiated treatments or investigations within 30 days, n (%)	465 (23.5)	672 (28.2)	513 (31.7)	18 (72)	<0.001
Endoscopy detecting GI bleeding within 7 days, n (%)	22 (1.1)	13 (0.5)	6 (0.4)	0 (0)	0.036
Acute myocardial infarction within 30 days, n (%)	1 (0.1)	3 (0.1)	42 (2.6)	0 (0)	<0.001
Ventricular tachycardia within 30 days, n (%)	0 (0)	5 (0.2)	13 (0.8)	1 (4)	<0.001
Cardiac arrest within 30 days, n (%)	5 (0.2)	4 (0.2)	6 (0.4)	1 (4)	0.002
Stroke within 30 days, n (%)	11 (0.6)	23 (1.0)	22 (1.4)	2 (8)	<0.001
Transitory Cerebral Ischemia within 30 days, n (%)	5 (0.2)	14 (0.6)	7 (0.4)	0 (0)	0.406
Chronic obstructive pulmonary disease requiring non invasive ventilation within 7 days, n (%)	1 (0.1)	12 (0.5)	27 (1.7)	3 (12)	<0.001
Transfer to another hospital (apart for rehabilitation) within the current hospitalization, n (%)	62 (3.1)	90 (3.8)	188 (11.6)	7 (28)	<0.001
Admission to intensive care unit within 30 days, n (%)	5 (0.2)	22 (0.9)	47 (2.9)	12 (48)	<0.001
Admission to intermediate-intensive care unit within 14 days, n (%)	6 (0.3)	28 (1.2)	53 (3.3)	6 (24)	<0.001
Death within 30 days, n (%)	44 (2.2)	86 (3.6)	82 (5.1)	7 (28)	<0.001
Panel B: Events, which identifies a high-risk patient					
Admission to intensive care unit within 30 days or semi-intensive care unit within 14 days, n (%)	11 (0.5)	43 (1.8)	90 (5.6)	14 (56)	<0.001
Death within 30 days, n (%)	44 (2.2)	86 (3.6)	82 (5.1)	7 (28)	<0.001

*P value indicates significant difference between the triage categories. Green/yellow/orange/red refers to triage acuity level in DEPT: non-urgent/urgent/emergent/resuscitation respectively, (Table 1 for detalis)

There was a significant variation in reporting frequency across different investigators for only two events: trombolysis (0 %-1.2 %, *p* = 0.04) and admission to intensive care (0.8-3.6 %, *p* < 0.01).

### Characteristics of the patient admissions not included

There were 378 (5.9 %) patient admissions not included for further analysis, due to missing triage category and/or blood sample for the biobank. Compared to the included patients, they had a higher age, longer LOS, lower BP and SpO_2_ at admission, as well as higher HR and RR. Significantly more patients were triaged *red* or *orange* (35.4 % vs. 27.3 %). Many events were more common in this group: i.v. treatment, i.v.-diuretics, antibiotic treatment, admissions lasting more than 3 days, not due to social factors or complications due or to initiated treatments or investigations within 30 days, cardiac arrest within 30 days, admission to intensive or semi-intensive care and death within 30 days. They also had a significantly higher burden of comorbidities such as diabetes, kidney disease, liver disease, rheumatic disease, use of alcohol and smoking. They were more often living alone, residents of a nursing home or subjects to domestic help. (All data including *p*-values are presented in Additional file 2: Table S2)

There were no events in 106 (28 %) of the patient admissions not included corresponding to 3.8 % of the total number of admissions with no events.

### Missing data

Presenting complaint or admission diagnosis was lacking in the patient file in <2 % of cases (Figs. 2 and 3), and all 8 routine laboratory tests were available in 5548 admissions (92.4 %). BP, HR and SpO_2_ were all available in 84 % of admissions. The most frequently missing variables from the triage were temperature (27.4 % missing) and respiratory rate (18.4 % missing) (Additional file 1: Table S1).

## Discussion

We achieved the goal of assembling a database with data and blood samples from more than 6000 admissions of consecutive patients admitted to the hospital through the emergency department. Patients were included 24 h a day, 7 days a week, which resulted in inclusion of 94.1 % of all admitted patients.

Crowding in the emergency department can be detrimental to patient outcome [2, 3] and with economical cutbacks in the health sector, prioritizing resources is crucial. A recent Danish study based on 2.65 million patient admissions shows that a high bed occupancy significantly increases in-hospital mortality and 30-day mortality [4]. In a recent article by O’Cathain et al., 22 % of almost 15 000 000 acute emergency admissions were deemed potentially unnecessary based on review of the admission diagnoses, searching for conditions know to be rich in avoidable admissions [5]. However, early identification of low-risk patients in the emergency department can be challenging. ER crowding could potentially be alleviated by faster discharge and/or faster admission to an inpatient bed.

The use of clinical scores for risk prediction have previously shown benefit primarily in groups of patients and not individuals [23], but even though previous models have not had the diagnostic quality to apply on individuals it is still essential to retest this idea in a more modern and advanced model. Future studies will show if the primary aim of the TRIAGE study can be fulfilled, but if it is possible to develop a model with the desired sensitivity and specificity of unnecessary admissions, this could be a potential major aid to the physicians in the ED.

There is considerable potential in using biochemichal markers in prognostic staging, also as a tool for identifying low-risk patients [17], and already it has been proven that combinations of vital values and standard blood tests have a high discriminatory power between high and low-risk patients in the acute setting [24, 25]. However, it has also been shown that the standard blood panel have only limited predictive power in risk assessment when added to physiological parameters [26], future studies can reveal whether prognostic information can be significantly improved using a whole panel of new and old inflammatory biomarkers. Several biomarkers have shown prognostic strength, for example C-reactive protein, suPAR, YKL-40, Pentraxin-3, Copeptin and Troponin T [11–16].

Many patients (44.2 %) in the TRIAGE database experienced no predefined ‘events’ during admission and hence were not subject to treatment that absolutely necessitated hospitalization. However, some admissions might still have been necessary. Therefore, any patient admission file with no events was scrutinized by medical experts to discover if the admission could be considered necessary. We found a significant relationship between triage category and increasing age, male gender, comorbidities and many predefined events. However, since there were equal amounts of patients with no ‘events’ in the green, yellow and orange triage categories (Table 3), the DEPT triage model seems inappropriate for predicting the outcome of these patients with lower risk.

### Strengths and limitations

An optimal score in an emergency department should be applicable in all patients as diagnoses might not be clear at patient arrival. However it is possible that the score varies between diagnoses and we have therefore planned subgroup analyses for the different disease categories. Patient inclusion covers many subgroups within internal medicine, surgery and neurology, which is significant strenght giving that the optimal score should be applicable in all patients. The current form of DEPT triage staging has been performed at the hospital for several years and was completely implemented and running steadily thoughout the study period. Earlier medical history was assessed by the patient file and not by questioning, which minimizes reporting bias. We had access to entries in the patient file even after eventual transfer to another hospital. Several investigators participated in extrating the events from the patient files, but each file was assessed by only one investigator and interobserver variability was not adressed. However, the frequency of reporting of events was similar across all investigators except for two events (trombolysis and admission to intensive care) and the differences were very small in absolute numbers, because few patients in the database encountered these two events. This variation is likely coincidence. We are aware that false measurements of vital signs are likely to have occurred as it would in normal clinical practice, but we have not evaluated interobserver agreement between the nurses who performed the measurements.

Nearly 6 % of admissions were not included and this constitutes a selection bias. Furthermore, there were few included patients in the ‘red’ triage group (0.4 %). Therefore, we cannot extrapolate the biomarker levels in the red triage group into a general Danish high-risk population. A comparative analysis shows, that the non-included patients were generally more ill. It was evident during data collection, that it was often in the most acute patients that no additional blood sample was taken for the biobank. Additionally; in the capital region of Denmark, seriously injured patients are brought directly to a tertiary trauma centre in Copenhagen, which also means that hyperacute patients are underrepresented in the TRIAGE database. This means our final model will have serious limitations in identifying trauma patients. However importantly, only 3.8 % of the total admitted patients with no ‘events’ are missing from the TRIAGE database, which probably makes subsequent biomarker levels valid for the general population of low-risk patients admitted to a Danish hospital, keeping in mind that patients with blue triage category (minor complaints and injuries) are not included in the TRIAGE database as they did not fulfill our criteria of admission in the study. Finally, the TRIAGE database is limited since we did not record the precise time the patients spend in the ER before discharge or admission to an inpatient bed. However, these data are not needed to construct the TRIAGE algorithm, since we use ‘events’ as measurements of outcome. In future studies, when the TRIAGE model could be implemented in the clinic, we have to record ER admission time to conclude if the model has any positive impact on ER crowding as well as the cost effectiveness of the model.

### Time schedule and status

At present time, the database has been completed and further data processing (including the identification of unnecessary hospitalizations) will take place during 2015. Analysis of the biomarkers is on-going and is expected to be finished during 2015.

## Conclusions

The TRIAGE database has been completed and includes data and blood samples from 6005 unselected consecutive hospitalized patients, corresponding to 94.1 % of all admissions over a three month period. More than 40 % experienced no events and were therefore potentially unnecessary hospital admissions.

## Additional files

Additional file 1: Table S1. Vital values according to Triage stage. (DOCX 120 kb) Additional file 2: Table S2. Characteristics of the included and not included admissions. (DOCX 127 kb)

## Abbreviations

ED: Emergency department
LOS: Lenght of stay
COPD: Chronic obstructive pulmonary disease
SuPAR: Soluble urokinase plasminogen activator receptor
DEPT: Danish emergency process triage
ADAPT: Adaptive process triage
BP: Blood pressure
HR: Heart rate
RR: Respiratory rate
SpO_2_: Saturation of peripheral oxygen
Tp: Temperature
SD: Standard deviation
CI: Confidence interval
