# Supplementary material for: The formation and design of the TRIAGE study - baseline data on 6005 consecutive patients admitted to hospital from the emergency department
Source: Scand J Trauma Resusc Emerg Med. 2015 Dec 1;23:106. doi: 10.1186/s13049-015-0184-1 (PMC4667414; doi:10.1186/s13049-015-0184-1)
Supplement: Additional file 1: Table S1. — Vital values according to Triage stage. (DOCX 120 kb) [file 13049_2015_184_MOESM1_ESM.docx]

|  | Green (n=1978) | Yellow (n=2386) | | Orange (n=1616) | Red (n=25) | P value* |
| --- | --- | --- | --- | --- | --- | --- |
| Systolic BP (mmHg)  Mean (SD)  >140 n(%)  90-139 n(%)  <90 n(%)  Missing | 139 (23.6)  759 (35.8)  954 (48.2)  13 (0.7)  252 (12.7) | 135 (23)  788 (33.0)  1215 (50.9)  31 (1.3)  351 (14.7) | 135 (25.4)  564 (34.9)  839 (51.9)  36 (2.2)  177 (11.0) | | 141 (41.8)  9 (36.0)  11 (44.0)  3 (12.0)  2 (8.0) | <0.001 |
| HR (min^-1^)  Mean (SD)  >120 n(%)  50-119 n(%)  <50 n(%)  Missing | 80 (15.8)  23 (1.2)  1673 (84.6)  21 (1.1)  261 (13.2) | 83 (18.0)  78 (3.3)  1920 (80.5)  30 (1.3)  358 (15.0) | 84 (23)  117 (7.2)  1279 (79.1)  39 (2.4)  181 (11.2) | | 107 (32)  8 (32.0)  13 (52.0)  0 (0)  4 (16.0) | <0.001 |
| RR (min^-1^)  Mean (SD)  >18 n(%)  10-18 n(%)  <10 n(%)  Missing | 17 (3.2)  308 (15.6)  1299 (65.7)  12 (0.7)  357 (18.0) | 17 (4.1)  496 (20.8)  1410 (59.1)  16 (0.7)  464 (19.4) | 18 (4.6)  438 (27.1)  886 (54.8)  12 (0.7)  280 (17.3) | | 26 (9.7)  15 (60.0)  5 (20.0)  0 (0)  5 (20.0) | <0.001 |
| SpO_2_ (%)  Mean (SD)  95-100 n(%)  90-94 n(%)  <90 n(%)  Missing | 97 (2.3)  1544 (78.1)  131 (6.6)  21 (1.1)  282 (14.3) | 97 (2.1)  1753 (73.5)  206 (8.6)  41 (1.7)  386 (16.2) | 97 (2.8)  1241 (76.8)  138 (8.5)  31 (1.9)  206 (12.7) | | 94 (4.6)  16 (64)  3 (12.0)  4 (16.0)  2 (8.0) | <0.001 |
| Tp (°C)  Mean (SD)  >40 n(%)  38.1-40 n(%)  36.0-38.0 n(%)  <36 n(%)  Missing | 36.8 (0.8)  6 (0.3)  58 (2.9)  1165 (58.9)  196 (9.9)  553 (28.0) | 36.8 (0.8)  5 (0.2)  110 (4.6)  1337 (56.0)  222 (9.3)  712 (29.8) | 36.6 (0.8)  5 (0.2)  50 (3.1)  759 (47.0)  215 (13.3)  587 (36.3) | | 36.7 (0.7)  0 (0)  0 (0)  7 (28.0)  1 (4.0)  17 (68.0) | <0.001 |

**Supplementary table S1 - Vital values according to Triage stage**

*P value indicates significant difference between the triage categories. BP: Blood pressure, HR: Heart rate, RR: Respiratory Rate, SpO_2_: peripheral arterial oxygen saturation, Tp: Temperature, SD: standard deviation. Green/yellow/orange/red refers to triage acuity level in DEPT: non-urgent/urgent/emergent/resuscitation respectively, (table 1 for detalis).
