# Supplementary material for: The formation and design of the TRIAGE study - baseline data on 6005 consecutive patients admitted to hospital from the emergency department
Source: Scand J Trauma Resusc Emerg Med. 2015 Dec 1;23:106. doi: 10.1186/s13049-015-0184-1 (PMC4667414; doi:10.1186/s13049-015-0184-1)
Supplement: Additional file 2: Table S2. — Characteristics of the included and not included admissions. (DOCX 127 kb) [file 13049_2015_184_MOESM2_ESM.docx]

**Supplementary table S2 - Characteristics of the included and not included admissions**

|  | Included (n=6005) | Not included (n=378) | P value* |
| --- | --- | --- | --- |
| Age, median years (IQR) | 62 (44-76) | 68 (47-89) | <0.001 |
| Gender, male n(%) | 2992 (49.8) | 171 (45.2) | 0.084 |
| LOS, median days (IQR) | 1 (4) | 2 (5) | <0.001 |
| Red or Orange triage category**, n(%) | 1641 (27.3) | 101 (35.4) | <0.001 |
| Ischemic heart disease n(%) | 587 (9.8) | 35 (9.3) | 0.743 |
| Heart failure, n(%) | 348 (5.8) | 26 (6.9) | 0.384 |
| Hypertension, n(%) | 1337 (22.3) | 82 (21.7) | 0.795 |
| Diabetes, n(%) | 628 (10.5) | 54 (14.3) | 0.019 |
| COPD, n(%) | 491 (8.2) | 41 (10.8) | 0.069 |
| Kidney disease, n(%) | 211 (3.5) | 22 (5.8) | 0.020 |
| Liver disease, n(%) | 100 (1.7) | 24 (6.3) | <0.001 |
| Rheumatic disease, n(%) | 129 (2.1) | 14 (3.7) | 0.047 |
| Cancer, n(%) | 831 (13.8) | 62 (16.4) | 0.163 |
| Smoking, n(%):  Former  Current | 1333 (22.2)  1454 (24.2) | 70 (18.5)  115 (30.4) | <0.001 |
| Alcohol abuse, n(%) | 511 (8.5) | 54 (14.3) | <0.001 |
| Social aspects, n(%):  Living alone  Domestic help  Nursing home | 1254 (20.9)  547 (9.1)  321 (5.3) | 97 (25.7)  71 (18.8)  43 (11.4) | 0.027  <0.001  <0.001 |
| Systolic BP, mean SD (mmhg) | 136 (24.2) | 129 (26.9) | <0.001 |
| HR, mean SD (min^-1^) | 82 (19.0) | 90 (15.2) | <0.001 |
| RR, mean SD (min^-1^) | 17 (4.0) | 19 (5.2) | <0.001 |
| SpO_2_, mean SD (%) | 97 (2.6) | 96 (4.4) | <0.001 |
| Tp, mean SD (°C) | 36.8 (0.8) | 36.8 (1.0) | 0.064 |
| No events, n(%) | 106 (28.0) | 2654 (44.2) | <0.001 |
| Acute surgery, n(%) | 206 (3.4) | 12 (3.2) | 0.790 |
| Surgery during admission, n(%) | 377 (6.3) | 30 (7.9) | 0.201 |
| Non-scheduled surgery within 14 days, n(%) | 172 (2.0) | 7 (1.9) | 0.810 |
| Trombolysis, n(%) | 28 (0.5) | 3 (0.8) | 0.375 |
| Endovascular procedure, n(%) | 233 (3.9) | 11 (2.9) | 0.340 |
| Antibiotics > 24 hours within 7 days, n(%) | 1235 (20.6) | 136 (36.0) | <0.001 |
| i.v.diuretics > 1 times within 14 days, n(%) | 205 (3.4) | 32 (8.5) | <0.001 |
| Other i.v.-treatment > 1 times within 14 days, n(%) | 1233 (20.5) | 146 (38.6) | <0.001 |
| Admissions lasting more than 3 days, not due to social factors or complications due or to initiated treatments or investigations within 30 days, n(%) | 1668 (27.8) | 157 (41.5) | <0.001 |
| Endoscopy detecting GI bleeding within 7 days, n(%) | 41 (0.7) | 5 (1.3) | 0.154 |
| Acute myocardial infarction within 30 days, n(%) | 46 (0.8) | 0 (0) | 0.088 |
| Ventricular tachycardia within 30 days, n(%) | 19 (0.3) | 2 (0.5) | 0.484 |
| Cardiac arrest within 30 days, n(%) | 16 (0.3) | 6 (1.6) | <0.001 |
| Stroke within 30 days, n(%) | 58 (1.0) | 7 (1.9) | 0.096 |
| Transitory Cerebral Ischemia within 30 days, n(%) | 26 (0.4) | 1 (0.3) | 0.625 |
| Chronic obstructive pulmonary disease requiring non invasive ventilation within 7 days, n(%) | 43 (0.7) | 5 (1.3) | 0.185 |
| Transfer to another hospital (apart for rehabilitation) within the current hospitalization, n(%) | 347 (5.8) | 19 (5.0) | 0.525 |
| Admission to intensive care unit within 30 days, n(%) | 86 (1.4) | 19 (5.0) | <0.001 |
| Admission to intermediate-intensive care unit within 14 days, n(%) | 93 (1.5) | 25 (6.6) | <0.001 |
| Death within 30 days, n(%) | 219 (3.6) | 50 (13.2) | <0.001 |

*P value indicates significant difference between the triage categories. ** Some (n=89) of the not included admissions were due to missing triage information and therefore this row depends only on the patients missing due to lack of blood sample (n=289). Alcohol abuse was defined as ≥ 3 units of alcohol/day.

LOS: Lenght Of Stay, COPD: Chronic Obstructive Pulmonary Disease, BP: Blood pressure, HR: Heart rate, RR: Respiratory Rate, SpO_2_: peripheral arterial oxygen saturation, Tp: Temperature, SD: standard deviation
